# Age-related sex differences in the expression of important disease-linked mitochondrial proteins in mice

**DOI:** 10.1186/s13293-019-0267-1

**Published:** 2019-12-05

**Authors:** Michael Moschinger, Karolina E. Hilse, Anne Rupprecht, Ute Zeitz, Reinhold G. Erben, Thomas Rülicke, Elena E. Pohl

**Affiliations:** 1 Institute of Physiology, Pathophysiology and Biophysics, Department of Biomedical Sciences, University of Veterinary Medicine, Vienna, Austria; 2 Institute of Pharmacology and Toxicology, Rostock University Medical Center, Rostock, Germany; 3 Institute of Laboratory Animal Science, Department of Biomedical Sciences, University of Veterinary Medicine, Vienna, Austria

## Abstract

**Abstract:**

The prevalence and progression of many illnesses, such as neurodegenerative and cardiovascular diseases, obesity, and cancer, vary between women and men, often in an age-dependent manner. A joint hallmark of these diseases is some type of mitochondrial dysfunction. While several mitochondrial proteins are known to be regulated by sex hormones, the levels of those proteins have not been systematically analyzed with regard to sex and age, and studies that consider sex and/or age differences in the protein expression are very rare. In this study, we compared the expression patterns of physiologically important mitochondrial proteins in female and male C57BL/6N mice of age cohorts frequently used in experiments. We found that sex-related differences in the expression of uncoupling proteins 1 and 3 (UCP1 and UCP3) occur in an age-dependent manner. The sex-specific expression of UCP1 and UCP3 in brown adipose tissue (BAT) was inversely correlated with differences in body weight. Expression of UCP4 in the brain, Complex I in the spleen, and Complex II in the brain and BAT was least affected by the sex of the mouse. We further demonstrated that there are serious limitations in using VDAC1 and actin as markers in western blot analyses, due to their sex- and age-specific fluctuations. Our results confirm that sex and age are important parameters and should be taken into account by researchers who examine the mechanistic aspects of diseases.

**Highlights:**

I. The levels of UCP1 and UCP3 protein expression differ between females and males in an age-dependent manner.
II. Pre-pubertal expression of almost all proteins tested in this study does not depend on the sex of the mouse.
III. Expression of VDAC1 and actin, which are often used as loading control proteins in western blot analysis, is tissue-specifically influenced by sex and age.

## Introduction

In recent years, sex-based differences have become more apparent in the pathogenesis, progression, and treatment outcomes of various human diseases, including diabetes, obesity, and cardiovascular disease, as well as autoimmune and neurological dysfunction [1, 2]. The factors thought to contribute to these sex-based differences in pathophysiology of various diseases are related to sex chromosomes, miRNAs, different levels of circulating steroid hormones (estrogen, androgens, and progesterone), nutrition, microbiota, and anatomical differences [3–9]. The global expression of sex hormone receptors in cells suggests that their influence on gene expression is higher than previously assumed [4]. However, biomedical research is often biased because potential sex differences are not accounted for in a study’s design and data analysis [10]. Sex bias is especially prominent in the field of neuroscience, due to the prevalent assumption that sex does not influence the physiology of the nervous system [11, 12].

Sex hormone receptors (e.g., estrogen receptors) are localized in the mitochondria of certain cells and influence mitochondrial physiology [13]. Additionally, it was reported that sex hormones can affect the expression of mitochondrial proteins encoded by either mitochondrial (mtDNA) or nuclear DNA [14, 15]. mtDNA is exclusively inherited from a mother in most mammals. Several mitochondrial proteins, especially mitochondrial outer membrane protein voltage-dependent anion channel 1 (VDAC1), are often used as a quantitative control in western blot (WB) analyses [16, 17]. However, their sex- and age-specific expression has been poorly investigated, and the lack of knowledge in this area can lead to erroneous conclusions in various studies. Earlier, an age-dependent decrease in the expression of cytosolic proteins that are routinely used as loading controls, such as glyceraldehyde 3-phosphate dehydrogenase (GAPDH), α-tubulin, and β-actin, in skeletal muscles (SkM) was already reported [18], suggesting that these proteins might be altered in an age-dependent manner in human tissues.

The mouse is a popular model for studying human diseases because (i) of its small size and rapid reproduction, (ii) its genome shares 85% identity with the human genome [19], (iii) many of its physiological systems function in manner similar to those in humans, and (iv) loss- and gain-of-function mutations are relatively easy to introduce into the mouse genome [20]. The genotype and phenotype of the inbred strain C57BL/6N have been well-characterized and the strain is frequently used as background for the generation of genetically modified mouse models. Sex-specific factors such as fluctuating sex hormone levels in female mice and strong fighting behavior in male mice often lead to a decision to use only one sex in biomedical studies [11].

Here, we examined the influence of sex on the expression of essential mitochondrial proteins in C57BL/6N mice at different life stages. We investigated three main protein groups that included (i) respiratory chain proteins Complex I (CI), Complex II (CII), and ATP synthase (CV), (ii) mitochondrial uncoupling proteins (UCP1-UCP4), and (iii) VDAC1 in the brain, SkM, brown adipose tissue (BAT), and spleen. Additionally, we compared the expression of two cytosolic proteins (α- and β-actin), which are often used as loading controls in WB analyses.

## Materials and methods

### Animals and standard preparation

Female and male C57BL/6NRj (B6) mice (ages 1, 5, and 10 months) in SPF quality were housed in a rodent facility (photoperiod 12L/12D, temperature 22.0 °C ± 2.0 °C). Food and water were available ad libitum and the mice were fed with a commercial regular mouse diet (ssniff Spezialdiäten GmbH, Germany). Mice were maintained in groups in polycarbonate cages (Type IIL, Tecniplast, Italy) lined with bedding material (Lignocel®, heat treated, Rettenmaier KG, Austria) and enriched with nesting material (Pur-Zellin 4 × 5 cm; Paul Hartmann GmbH, Austria). For dissection and tissue sampling, the animals were weighed and then sacrificed by inhalation of a mixture of 79% CO_2_ and 21% O_2_ until breathing arrest, and then decapitated. For the quantification of all western blot results, tissue standards were produced from pooled (*n* = 10–20) B6 mice of different ages for each sex.

### Western blot analysis

WB analysis was performed as previously described [21, 22]. Briefly, samples of the brain, BAT, SkM (gastrocnemius muscle), and spleen tissue were homogenized in RIPA buffer (50 mM Tris; 150 mM sodium chloride; 1 mM EDTA; 1% sodium deoxycholate; 1% Triton X-100; 0.1% sodium dodecyl sulfate; pH 7.4) supplemented with a protease inhibitor cocktail (Sigma-Aldrich). The homogenates were then sonicated and centrifuged at 1000*g* for 10 min at 4 °C. Next, the supernatants were centrifuged again at 2.500*g* for 10 min at 4 °C. The total protein concentration was determined using a Pierce™ BCA Protein Assay Kit (Thermo Scientific, Waltham, MA, USA). Twenty micrograms for brain tissue and BAT and 50 μg for spleen and SkM (gastrocnemius muscle, GMsc) tissue were separated by SDS-PAGE. The separated protein bands were then transferred onto membranes that were incubated overnight at 4 °C with primary antibodies against UCP1 (1:1000, U6382, Sigma-Aldrich, Additional file 1: Figure S1A), UCP2 (evaluated in [23] Additional file 1: Figure S1B), UCP3 (evaluated in [21], Additional file 1: Figure S1C), UCP4 (evaluated in [24], Additional file 1: Figure S1D), subunit NDUFA9 of CI (1:3000, 459100, Invitrogen, Additional file 1: Figure. S2A), subunit SDHA of CII (1:7500, Ab14715, Abcam, Additional file 1: Figure. S2B), subunit beta of ATP synthase (1:5000, A21351, Invitrogen, Additional file 1: Figure S2, C), VDAC1 (1:5000, Ab14734, Abcam, Additional file 1: Figure S2D), α-actin (1:5000, Ab88226, Abcam, Additional file 1: Figure S2, E), and β-actin (1:10000, A5441, Sigma-Aldrich, Additional file 1: Figure S2, F); after which, the membranes were incubated with a secondary antibody that was conjugated with horseradish peroxidase. The immunostained protein bands were detected by chemiluminescence, and intensity of staining was quantified by using Launch Vision Works LS software. Examples of whole WB images are shown in Additional file 1: Figures S1 and S2 illustrating the protein bands against a protein ladder. The relative amount of each protein was calculated as the ratio of the staining intensity of the target protein band vs. the loaded standard band.

### Statistical analysis

Data from WB analyses are presented as the mean value ± SD of results obtained from at least two independent experiments (samples from 6 mice per sex and age). All data were analyzed using the two-sided *t* test and the two-sided one-way ANOVA test, and *p* values < 0.05 were considered to be statistically significant. The symbols * and #, ** and ##, or *** and ### indicate statistically significant *p* values of < 0.05, < 0.01, and < 0.001, respectively.

## Results

### Influence of sex and age on the expression of key mitochondrial proteins in the brain tissue

**Fig. 1:**
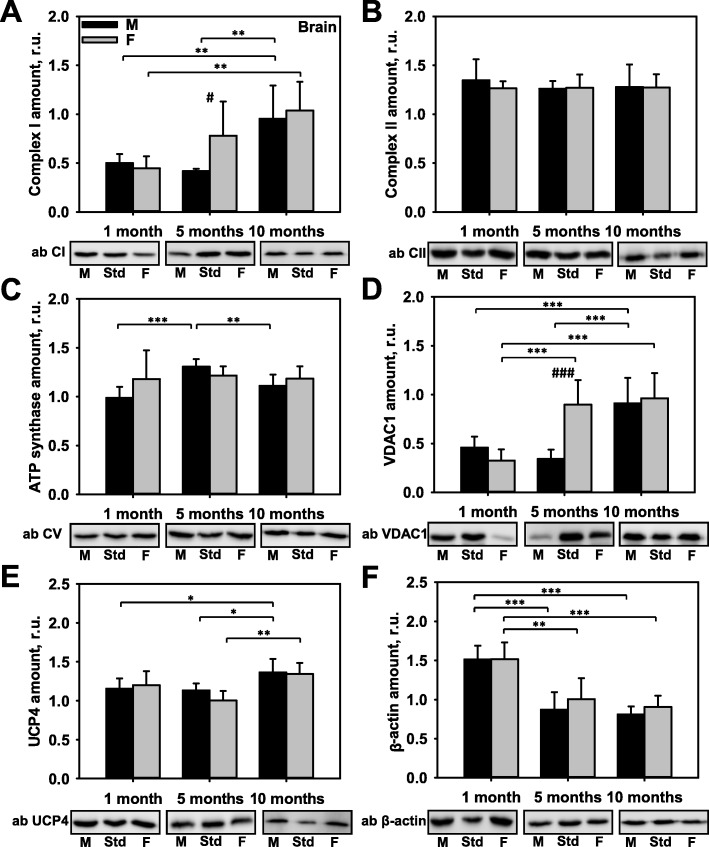
Comparison of protein expression in the brain tissues of female and male mice. Quantitative analysis of western blot (WB) images obtained from 1, 5, and 10-month-old female (F) and male (M) mice showing the relative amounts of Complex I (**a**), Complex II (**b**), ATP synthase (**c**), VDAC1 (**d**), UCP4 (**e**), and β-actin (**f**) as compared to a brain tissue standard (Std). Representative WB images are shown below the plots. 20 μg of total protein were loaded in each lane. Values represent the means ± SD of data obtained from six animals per group; *p < 0.05, **p < 0.01, and ***p < 0.001 (mark age differences); #*p* < 0.05, ##*p* < 0.01, and ###*p* < 0.001 (mark sex differences)

To evaluate whether the distribution of physiologically relevant mitochondrial proteins in the brain tissue differed between females and males, we evaluated the protein levels of UCP4; electron transport chain (ETC) proteins CI, CII, and CV; outer mitochondrial membrane protein VDAC1, and non-mitochondrial protein β-actin in pre-pubertal (1-month-old), pubertal (5-month-old), and post-pubertal (10-month-old) B6 mice of different sex at physiological conditions. Our results showed that all tested proteins were expressed at similar levels in male and female pre-pubertal mice (Fig. 1a–f). CII, ATP synthase, and UCP4 (Fig. 1b, c, e) did not show sex-specific expression at any of the life stages. CI, CV, VDAC1, UCP4, and β-actin were influenced by age. The greatest variations were found for CI (Fig. 1a; Additional file 1: Figure S4A) and VDAC1 (Fig. 1d and Additional file 1: Figure S4B) protein levels, and those two proteins showed similar expression patterns. Five-month-old female mice exhibited two-fold higher levels of CI and VDAC1 expression when compared with male mice (Table 1). In addition, both of those proteins displayed two-fold increases in expression from childhood to maturity in both sexes. Surprisingly, the level of β-actin expression (Fig. 1f) in post-pubertal mice declined by 50% when compared to β-actin expression in pre-pubertal mice, and the decline was not sex-specific.

**Table 1:**
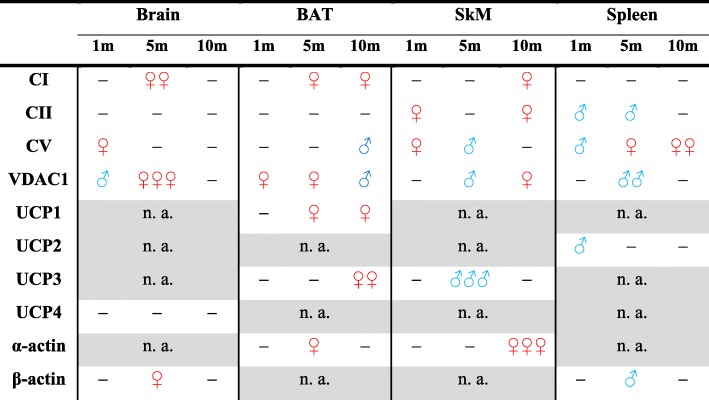
Summary of sex differences in the protein expression of Complex I (CI), Complex II (CII), ATP synthase (CV), VDAC1, UCP1, UCP2, UCP3, UCP4, α-actin, and β-actin measured in the brain, brown adipose tissue (BAT), skeletal muscle (SkM), and spleen tissue. Protein expression was compared between female and male mice at an age of one (1 m), five (5 m) and ten months (10 m) and is presented as ratios female/male (red) or male/female (blue). n. a. stands for not applicable. “-” indicates no or very small difference (1 < R < 1.1) in expression between female and male mice; ♀ (♂)—for R between 1.2 and 1.5; ♀♀ (♂♂)—for R between 1.6 and 2.0; and ♀♀♀ (♂♂♂)—over 2.00

### Influence of sex and age on the expression of key mitochondrial proteins in BAT

**Fig. 2:**
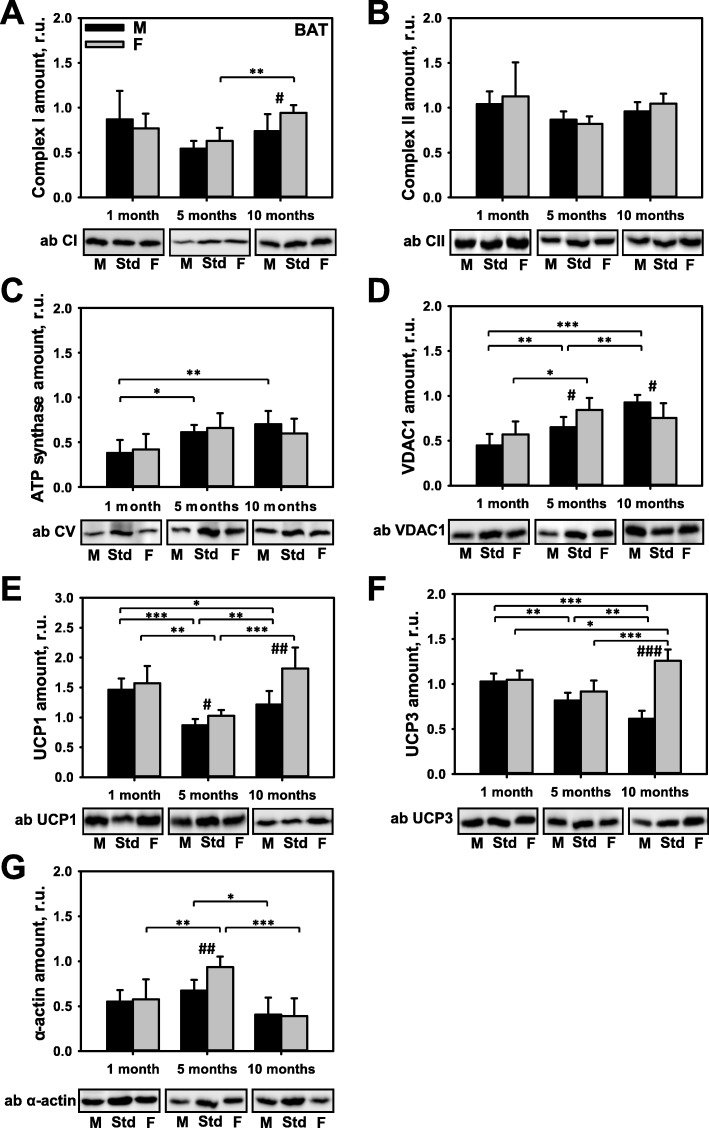
Comparison of protein expression in brown adipose tissue (BAT) from female and male mice. A quantitative analysis of western blot (WB) images obtained from 1, 5, and 10-month old female (F) and male (M) mice showing the relative amounts of Complex I (**a**), Complex II (**b**), ATP synthase (**c**), VDAC1 (**d**), UCP1 (**e**), UCP3 (**f**), and α-actin (**g**) as compared to a BAT tissue standard (Std). Representative WB images are shown below the plots. 20 μg of total protein were loaded in each lane. Values represent the means ± SD of data obtained from six animals per group; *p < 0.05, **p < 0.01, and ***p < 0.001 (mark age differences); #*p* < 0.05, ##*p* < 0.01, and ###*p* < 0.001 (mark sex differences)

BAT has been shown to be an important target for treating obesity [25, 26]. Because metabolic activity and non-shivering thermogenesis in BAT were described to be sex-specific [27, 28], we compared the expression levels of certain mitochondrial proteins (ETC complexes, VDAC1, UCP1, and UCP3) and a cytoskeletal protein (α-actin). Our results confirmed that all those proteins were similarly expressed in pre-pubertal mice (Fig. 2a–g). Furthermore, we did not detect any sex differences in the levels of CII and ATP synthase expression in post-pubertal mice (Fig. 2b, c). An age-dependent increase in VDAC1 expression coupled with a sex dimorphism was observed in 5- and 10-month-old mice (Fig. 2d). However, this sex dimorphism most greatly affected the levels of UCP1 and UCP3 expression in the 10-month-old mice (Fig. 2e, f and Additional file 1: Figure S4C). A time course of UCP1 expression showed a decrease in 5-month-old mice, followed by an increase at 10 months, and the increase was 33% greater in female mice. The levels of UCP3 expression were also increased in female mice at the age of 10 months and were approximately two-fold higher than those in male mice. Surprisingly, UCP3 expression in male mice declined with age. The level of α-actin expression was increased by 33% in 5-month-old female mice (Fig. 2g).

### Influence of sex and age on the expression of key mitochondrial proteins in SkM

**Fig. 3:**
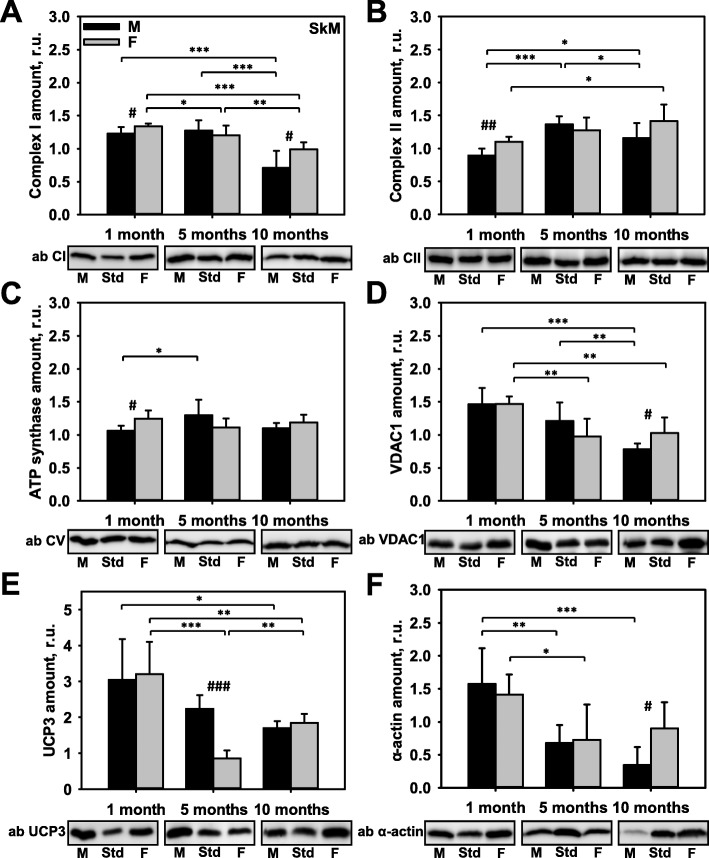
Comparison of protein expression in skeletal muscle (SkM) from female and male mice. A quantitative analysis of western blot (WB) images obtained from 1, 5, and 10-month old female (F) and male (M) mice showing the relative amounts of Complex I (**a**), Complex II (**b**), ATP synthase (**c**), VDAC1 (**d**), UCP3 (**e**), and α-actin (**f**) as compared to a SkM tissue standard (Std). Representative WB images are shown below the plots. 50 μg of total protein was loaded in each lane. Values represent the means ± SD of data obtained from six animals per group; *p < 0.05, **p < 0.01, and ***p < 0.001 (mark age differences); #*p* < 0.05, ##*p* < 0.01, and ###*p* < 0.001 (mark sex differences)

To evaluate whether metabolically relevant mitochondrial proteins in SkM displayed a sex-dependent pattern of expression, we measured the expression of mitochondrial ETC complexes, VDAC1, and UCP3, as well as a cytoskeletal protein (α-actin) as described above. Figure 3a–f demonstrates that the pre-pubertal expression of the measured proteins was similar in both sexes, with the exception that CII and ATP synthase showed slightly higher levels of expression in females than in males. While CII and ATP synthase exhibited hardly any sex-related differences at post-puberty, they did show age-dependent changes (Fig. 3b, c). CI showed a sex-specific decrease in both male and female mice at the age of 10 months (Fig. 3a). VDAC1 expression was sex-specific at the age of 10 months and showed the tendency to decline in both sexes (Fig. 3d). UCP3 abundance was strongly sex-dependent in 5-month-old animals and was three-fold higher in males (Fig. 3e). Male mice displayed an age-dependent decline in UCP3 expression, whereas UCP3 expression in females was decreased at the age of 5 months compared to 1-month-old mice and significantly increased again at the age of 10 months compared to the 5-month-old mice. An age-dependent decline in α-actin levels was also observed in both sexes but was more intense in males (Fig. 3f and Additional file 1: Figure S4D).

### Influence of sex and age on the expression of key mitochondrial proteins in the spleen

**Fig. 4:**
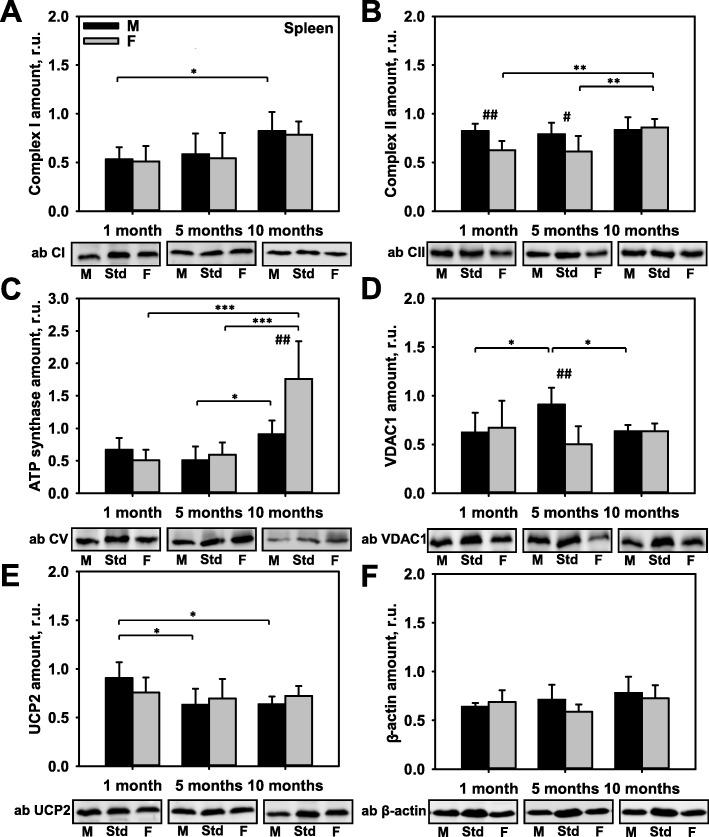
Comparison of protein expression in spleen tissue from female and male mice. A quantitative analysis of western blot (WB) images obtained from 1, 5, and 10-month old female (F) and male (M) mice showing the relative amounts of Complex I (**a**), Complex II (**b**), ATP synthase (**c**), VDAC1 (**d**), UCP2 (**e**), and β-actin (**f**) as compared to a spleen tissue standard (Std). Representative WB images are shown below the plots. 50 μg of total protein was loaded in each lane. Values represent the means ± SD of data obtained from six animals per group; *p < 0.05, **p < 0.01, and ***p < 0.001 (mark age differences); #*p* < 0.05, ##*p* < 0.01, and ###*p* < 0.001 (mark sex differences)

Immune response in the context of infectious diseases, cancer, and autoimmunity is known to be influenced by sex in an age-specific manner [2]. To test whether this phenomenon was reflected in the levels of various mitochondrial proteins, we evaluated the expression of CI, CII, ATP synthase, VDAC1, and UCP2, as well as expression of the cytoskeletal protein β-actin. Once again, the pre-pubertal levels of expression for all those proteins were similar in both sexes, with the exception of CII (Fig. 4a–f). Moreover, the post-pubertal expression patterns of CI, UCP2, and β-actin were similar in mice of both sexes (Fig. 4a, e, f). Surprising results were obtained for ATP synthase expression. Here, we observed a strong increase in ATP synthase levels at the age of 10 months, and the levels were two-fold higher in female mice (Fig. 4c and Additional file 1: Figure S4E). A sex dimorphism was detected in the expression pattern of VDAC1 in 5-month-old animals, which was two-fold higher in male mice (Fig. 4d). In contrast, the time course of VDAC1 expression in female mice remained constant over time.

## Discussion

When using standardized, wild-type B6 mice as an animal model, we found that the levels of UCP1 and UCP3 protein expression in male and female mice differed in an age-dependent manner, whereas the expression of UCP4 was similar in both sexes. Protein expression of ETC members has shown tissues-specific differences between males and females: CII and ATP synthases were similarly abandoned in the brain and BAT, CI expression was not influenced by sex and age in the spleen (see Table 1 for summary). We further demonstrated that VDAC1 and actin, which are regularly used as reference proteins in WB analyses, are tissue-specifically influenced by sex and age.

Few studies have investigated the sex-specific expression of proteins, and the existing studies have usually focused on only one tissue [11]. Moreover, in most studies, age groups are arbitrarily chosen, and only one age is usually tested for a specific condition. Here, we compared for the first time the expression patterns of physiologically important mitochondrial proteins in female and male B6 mice. To reflect a wide variety of scientific conditions, we tested frequently used age cohorts: 1, 5, and 10 months, respectively. These age choices reflect the physiologically important stages of murine life, such as pre-puberty, puberty, and adulthood. Our data showed that almost all the candidate proteins were expressed at similar levels in both sexes at a pre-pubertal age. However, significant sex differences become apparent in adulthood. Impressive sex dimorphisms were observed for two members of the mitochondrial uncoupling protein family, UCP1 and UCP3. These proteins showed a tendency for decreased expression with age in male mice, whereas, in females, we observed a decline in their expression at the age of 5 months, followed by an upregulation at 10 months. Several studies have provided evidence that the UCP family may be regulated by sex hormones [28–30]; however, their results are contradictory and/or based only on mRNA data or an analysis of one tissue type. It was previously shown that because of the unusually short half-life and strong post-translational regulation of UCP2 and UCP3 [31–33], a determination of their protein levels (and not mRNA levels) is the only accurate way to perform a functional analysis of these proteins [21, 23, 34]. Our results concerning UCP1 and UCP3 expression relate to age- and sex-dependent changes in body weight (Additional file 1: Figure S3).

The male mice in this study showed a significant increase in body weight with increasing age, whereas we observed only a slight increase in body weight of the female mice (Additional file 1: Figure S3). Notably, the female mice expressed higher levels of UCP1 and UCP3 in their BAT. Results obtained by using models of UCP3 gain-of-function and UCP3 loss-of-function have suggested that UCP3 helps protect against triglyceride accumulation in murine SkM [35, 36]. Therefore, the upregulation of UCP3 in females may prevent the occurrence of obesity-induced secondary disorders such as cardiovascular diseases and diabetes. The sex-specific expression of UCP1 and UCP3 could explain the different propensities of female and male rodents for becoming obese, and imply that a similar mechanism could exist in humans.

We observed that the protein expression of CI in the female brain was significantly higher at 5 months than in pre-pubertal and aged mice. In agreement with it, a study of Gaignard et al. (2015) showed that the NADH-linked respiration of isolated brain mitochondria was significantly higher in 3-month-old female mice compared to males and ovariectomized female mice [37]. Arias-Reyes et al. (2019) showed that the NADH-linked respiration in 3-months-old B6 mice is sex-dependent but the NADH-linked respiration in the brain stem and cortex was higher in male mice [38]. More in-depth research is needed to clarify the role of sex influences on the mitochondrial respiration and expression of ETC proteins in the different regions of the brain.

The question of whether VDAC1 undergoes sex dimorphisms is very important because in the positive case, it would detract from its often used as a reference protein. Here, we revealed for the first time that the post-pubertal levels of VDAC1 were affected by sex and age in all the analyzed tissues. In contrast, the levels of CII and ATP synthase expression were similar in female and male mice in the brain and BAT. This finding supports data that were obtained by an analysis of CI–CV genes in rats; namely no sexual dimorphism in genes that encode for proteins involved in oxidative phosphorylation in hearts of young and adult rats [39]. However, slight tissue-specific sex differences were detected in old mice. We found that ATP synthase was expressed at two-fold higher levels in the spleens of 10-month-old female mice; however, the expression was highly variable among the individual females tested (Additional file 1: Figure S4, E). It is possible that the deviations in ATP expression result from inflammatory processes (e.g., due to the activation of immune cells) that require different metabolic program [40].

Remarkably, we found that expression of the often-used cytoskeletal marker actin differed in the BAT, SkM, and brain tissues of males and females, and significantly declined with age. Our results support those reported in past studies concerning age-dependent decreases of β-actin in human SkM [18] and rat brain tissue [41]. It is important to emphasize that the use of conditionally expressed reference proteins for data normalization can result in different interpretations of study results. In one of our previous studies [42], the ratio of UCP4 to VDAC1 protein levels in murine brain tissue showed a decrease in UCP4 expression with increasing age, whereas the ratio of UCP4 to β-actin showed an increase in UCP4 expression. Both results were repeatable and verified in the current study (Additional file 1: Figure S5). However, the level of UCP4 expression was the same in all groups when it was not compared to a reference protein or normalized to an equally expressed loading control such as CII. This example convincingly demonstrates that the interpretation of experimental data obtained by using different housekeeping proteins can differ when factors such as sex and age are involved in a study. We identified CI (spleen), CII (brain and BAT), and UCP4 (brain) as the loading control proteins that showed the least degree of fluctuation (Table 1). Therefore, they could most likely serve as a reliable control in WB studies that compare different sex and/or age groups. β-actin showed a constant level of expression in the spleen tissue. CI, VDAC1, and α-actin displayed sex and age-specific variations in expression in most tissues. Thus, their use as loading controls should be carefully considered in experiments that compare sex or age groups of B6 mice.

## Significance and perspectives

The evaluation of sex- and age-dependent differences in the expression of several important mitochondrial proteins will help scientists consider sex or age dimorphisms in their future research. Our results highlight pronounced age-dependent differences in the expression of UCP1 and UCP3 that is especially important in the pathogenesis of obesity, diabetes, and cardiovascular diseases. Dissipating the excess of energy via uncoupling of the oxidative phosphorylation in brown adipocytes is a possible way to prevent overweight [43–45]. The nutritional environment and genetic background can activate the expression of UCPs in brite adipose tissue within white adipose tissue by adrenergic stimulation also in an adult organism. Understanding the regulation mechanism of UCP1/UCP3 and their exact role within the mitochondria inner membrane will provide new strategies to treat obesity and its related diseases.

## Supplementary information

**Additional file 1: Figure S1.** Whole WB images showing the expression of (A) UCP1 in BAT of one-month-old female (F1) and male (M1) mice; (B) UCP2 in the spleen of one-month-old female and male mice; (C) UCP3 in SkM of one-month-old female and male mice; and (D) UCP4 in the brain of five-month-old female and male mice compared to a tissue standard (Std). The protein ladder is illustrated on the left side of the images. The red rectangles mark the height of the individual protein bands **Figure S2.** Whole WB images showing the expression of CI (A), CII (B) and α-actin (E) in SkM of one-month-old female (F1) and male (M1) mice; and CV (C), VDAC1 (D) and ab β-actin (F) in brain of five-month-old female (F5) and male (M5) mice compared to a tissue standard (Std). The protein ladder is illustrated on the left side of the images. The red rectangles mark the height of the individual protein bands. Additional bands visible on the membranes are pre-stained proteins after stripping **Figure S3.** Body weight of one, five, and ten-months-old female (F) and male (M) mice. Values represent the means ± SD of data obtained from six animals per group; *** *p* < 0.001 **Figure S4.** Box plots diagrams to illustrate variable data points within the expression of (A) CI (from Fig. 1, A) and (B) VDAC1 (from Fig. 1, D) in brain; (C) UCP1 (from Fig. 2, E) in BAT; (D) α-actin (from Fig. 3, F) in SkM and (E) CV (from Fig. 4, C) in spleen of one, five and ten-months-old males (M1, M5, M10) and females (F1, F5, F10). The boundaries of the box represent 25%-75% of the values. The continuous line represents the median; and the dotted line represents the mean. The highest and lowest values are indicated by the whiskers **Figure S5.** Comparison of UCP4 protein expression in brain tissue from female and male mice. A quantitative analysis of WB images obtained from one, five, and ten-month old female (F) and male (M) mice showing the relative amounts of UCP4 normalized to VDAC1 (A) or normalized to β-actin (B). Values represent the means ± SD of data obtained from six animals per group; * *p* < 0.05, ** *p* < 0.01, and *** *p* < 0.001 (mark age differences); # *p* < 0.05 and ## *p* < 0.01 (mark sex differences)

## Data Availability

The datasets used and/or analyzed during the current study are available from the corresponding author on reasonable request.
