# Supplementary material for: Age-related sex differences in the expression of important disease-linked mitochondrial proteins in mice
Source: Biol Sex Differ. 2019 Dec 5;10:56. doi: 10.1186/s13293-019-0267-1 (PMC6896328; doi:10.1186/s13293-019-0267-1)
Supplement: Supplementary file 1 — Additional file 1: Figure S1. Whole WB images showing the expression of (A) UCP1 in BAT of one-month-old female (F1) and male (M1) mice; (B) UCP2 in the spleen of one-month-old female and male mice; (C) UCP3 in SkM of one-month-old female and male mice; and (D) UCP4 in the brain of five-month-old female and male mice compared to a tissue standard (Std). The protein ladder is illustrated on the left side of the images. The red rectangles mark the height of the individual protein bands Figure S2. Whole WB images showing the expression of CI (A), CII (B) and α-actin (E) in SkM of one-month-old female (F1) and male (M1) mice; and CV (C), VDAC1 (D) and ab β-actin (F) in brain of five-month-old female (F5) and male (M5) mice compared to a tissue standard (Std). The protein ladder is illustrated on the left side of the images. The red rectangles mark the height of the individual protein bands. Additional bands visible on the membranes are pre-stained proteins after stripping Figure S3. Body weight of one, five, and ten-months-old female (F) and male (M) mice. Values represent the means ± SD of data obtained from six animals per group; *** p < 0.001 Figure S4. Box plots diagrams to illustrate variable data points within the expression of (A) CI (from Fig. 1, A) and (B) VDAC1 (from Fig. 1, D) in brain; (C) UCP1 (from Fig. 2, E) in BAT; (D) α-actin (from Fig. 3, F) in SkM and (E) CV (from Fig. 4, C) in spleen of one, five and ten-months-old males (M1, M5, M10) and females (F1, F5, F10). The boundaries of the box represent 25%-75% of the values. The continuous line represents the median; and the dotted line represents the mean. The highest and lowest values are indicated by the whiskers Figure S5. Comparison of UCP4 protein expression in brain tissue from female and male mice. A quantitative analysis of WB images obtained from one, five, and ten-month old female (F) and male (M) mice showing the relative amounts of UCP4 normalized to VDAC1 (A) or normalized to β-actin (B). Val [file 13293_2019_267_MOESM1_ESM.pdf]

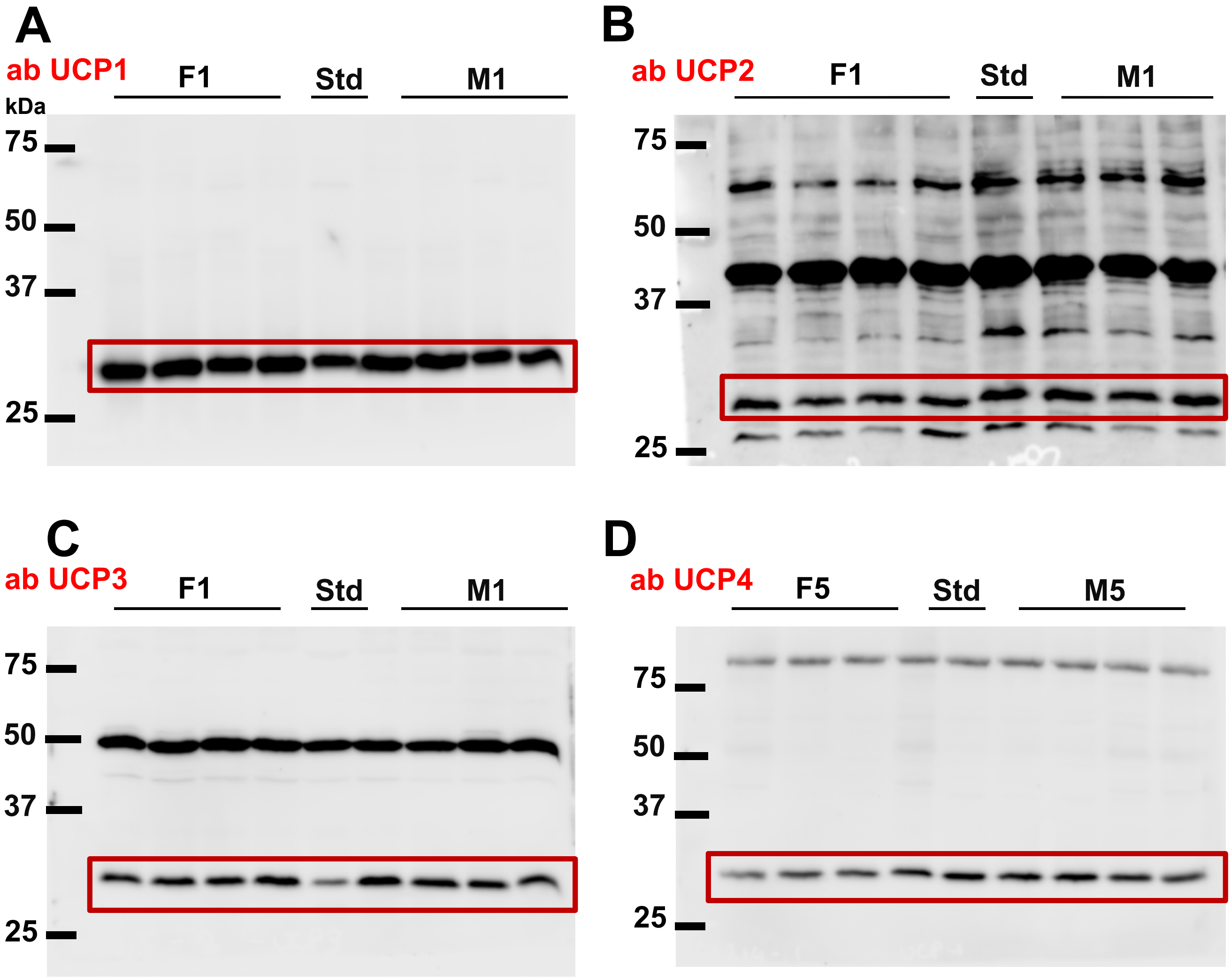

Supplementary figure S1. Whole WB images showing the expression of (A) UCP1 in BAT of one-month-old female (F1) and male (M1) mice; (B) UCP2 in the spleen of one-month-old female and male mice; (C) UCP3 in SkM of one-month-old female and male mice; and (D) UCP4 in the brain of five-month-old female and male mice compared to a tissue standard (Std). The protein ladder is illustrated on the left side of the images. The red rectangles mark the height of the individual protein bands.

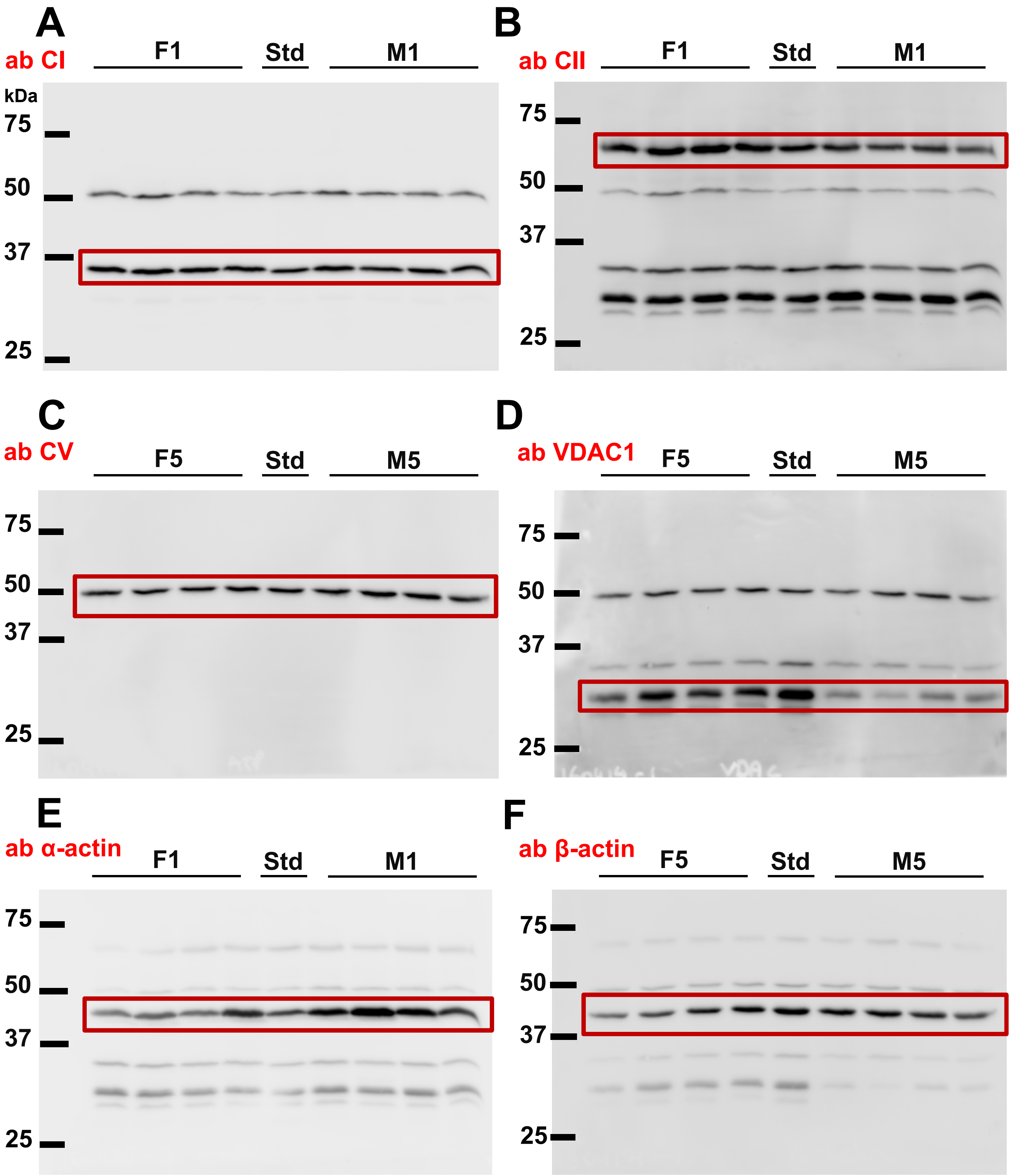

Supplementary figure S2. Whole WB images showing the expression of CI (A), CII (B) and α-actin (E) in SkM of one-month-old female (F1) and male (M1) mice; and CV (C), VDAC1 (D) and ab β-actin (F) in brain of five-month-old female (F5) and male (M5) mice compared to a tissue standard (Std). The protein ladder is illustrated on the left side of the images. The red rectangles mark the height of the individual protein bands. Additional bands visible on the membranes are pre-stained proteins after stripping.

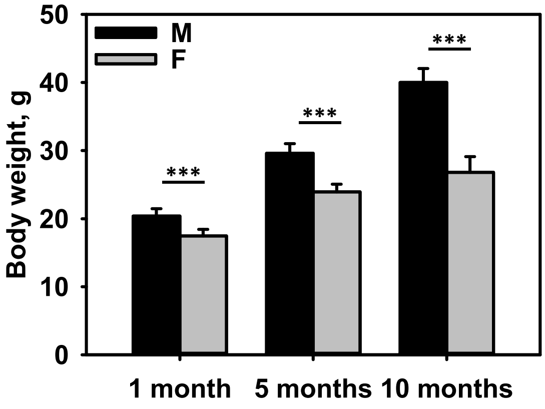

Supplementary figure S3. Body weight of one, five, and ten-months-old female (F) and male (M) mice. Values represent the means ± SD of data obtained from six animals per group; *** $p < 0.001$.

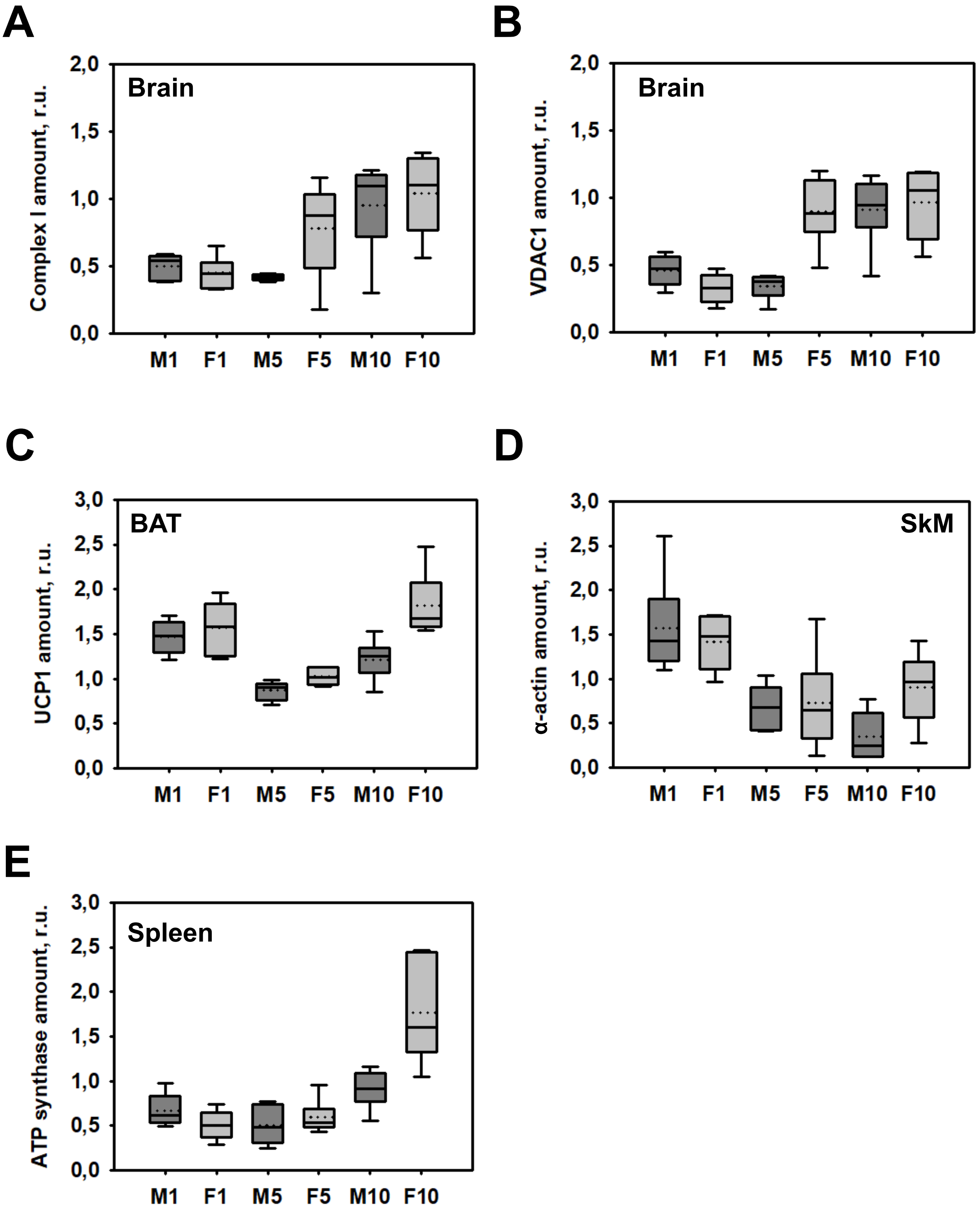

Supplementary figure S4. Box plots diagrams to illustrate variable data points within the expression of (A) CI (from Fig. 1, A) and (B) VDAC1 (from Fig. 1, D) in brain; (C) UCP1 (from Fig. 2, E) in BAT; (D) α-actin (from Fig. 3, F) in SkM and (E) CV (from Fig. 4, C) in spleen of one, five and ten-months-old males (M1, M5, M10) and females (F1, F5, F10). The boundaries of the box represent 25%-75% of the values. The continuous line represents the median; and the dotted line represents the mean. The highest and lowest values are indicated by the whiskers.

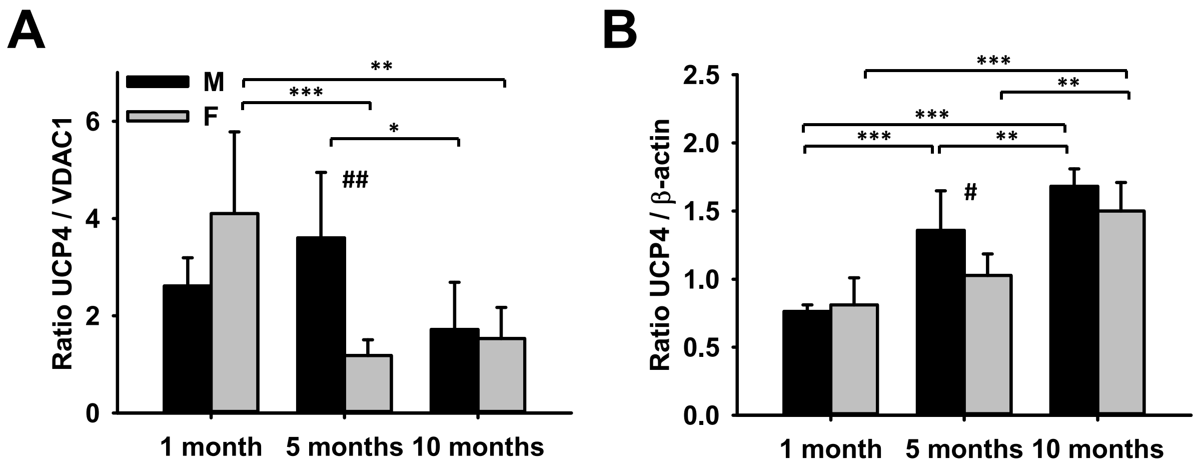

Supplementary figure S5. Comparison of UCP4 protein expression in brain tissue from female and male mice. A quantitative analysis of WB images obtained from one, five, and ten-month old female (F) and male (M) mice showing the relative amounts of UCP4 normalized to VDAC1 (A) or normalized to β-actin (B). Values represent the means ± SD of data obtained from six animals per group; * $p < 0.05$, ** $p < 0.01$, and *** $p < 0.001$ (mark age differences); # $p < 0.05$ and ## $p < 0.01$ (mark sex differences).
